# Mechanisms of Team-Sport-Related Brain Injuries in Children 5 to 19 Years Old: Opportunities for Prevention

**DOI:** 10.1371/journal.pone.0058868

**Published:** 2013-03-28

**Authors:** Michael D. Cusimano, Newton Cho, Khizer Amin, Mariam Shirazi, Steven R. McFaull, Minh T. Do, Matthew C. Wong, Kelly Russell

**Affiliations:** 1 Injury Prevention Research Office, St. Michael’s Hospital, University of Toronto, Toronto, Canada; 2 Division of Neurosurgery, St. Michael’s Hospital, University of Toronto, Toronto, Canada; 3 Health Surveillance and Epidemiology Division, Public Health Agency of Canada, Ottawa, Canada; California Pacific Medicial Center Research Institute, United States of America

## Abstract

**Background:**

There is a gap in knowledge about the mechanisms of sports-related brain injuries. The objective of this study was to determine the mechanisms of brain injuries among children and youth participating in team sports.

**Methods:**

We conducted a retrospective case series of brain injuries suffered by children participating in team sports. The Canadian Hospitals Injury Reporting and Prevention Program (CHIRPP) database was searched for brain injury cases among 5–19 year-olds playing ice hockey, soccer, American football (football), basketball, baseball, or rugby between 1990 and 2009. Mechanisms of injury were classified as “struck by player,” “struck by object,” “struck by sport implement,” “struck surface,” and “other.” A descriptive analysis was performed.

**Results:**

There were 12,799 brain injuries related to six team sports (16.2% of all brain injuries registered in CHIRPP). Males represented 81% of injuries and the mean age was 13.2 years. Ice hockey accounted for the greatest number of brain injuries (44.3%), followed by soccer (19.0%) and football (12.9%). In ice hockey, rugby, and basketball, striking another player was the most common injury mechanism. Football, basketball, and soccer also demonstrated high proportions of injuries due to contact with an object (e.g., post) among younger players. In baseball, a common mechanism in the 5–9 year-old group was being hit with a bat as a result of standing too close to the batter (26.1% males, 28.3% females).

**Interpretation:**

Many sports-related brain injury mechanisms are preventable. The results suggest that further efforts aimed at universal rule changes, safer playing environments, and the education of coaches, players, and parents should be targeted in maximizing prevention of sport-related brain injury using a multifaceted approach.

## Introduction

Participation in sport is a valuable contributor to physical and mental well-being [1]; however, involvement in many sports is also associated with an increased risk of brain injury [2]. This is particularly concerning for children and youth, who are at risk of long-term cognitive deficits following sports-related traumatic brain injury [3]. There is a relatively high rate of youth sport participation and sport-related brain injuries [4], and currently there is no definitive treatment to ensure complete recovery.

Existing literature concerning paediatric team sports head injury prevention is incomplete, as most is targeted towards a few sports, including ice hockey [4]–[8]. Furthermore, literature that includes other sports has not identified key mechanisms of injury [9], or if mechanisms were addressed, it has been limited to a single Canadian province [10], sport [11], or league/institution [12]. A better understanding of brain injury mechanisms across sports is needed. This study provides a comprehensive comparative analysis of injury mechanisms across different sports amongst a large population of children aged 5–19 years. The objective of this study was to provide descriptions of team sport brain injury mechanisms in Canadian children and youth.

## Methods

Ethical approval was granted by the Research Ethics Board at St. Michael’s Hospital, Toronto, Canada. This study utilized de-identified, administrative data and the ethics committee approved the waiver of consent.

In 1990, the Canadian Hospitals Injury Reporting and Prevention Program (CHIRPP) was developed as an emergency department (ED)-based injury surveillance system [13].CHIRPP currently operates in the EDs of 11 paediatric and 4 general Canadian hospitals. CHIRPP provides information such as what the patient was doing at the time of injury, injury cause, when and where the injury occurred, and the age and sex of the patient. ED staff report open-ended details regarding the nature of injury and fixed-choice details on the injured body part and treatment. The validity of CHIRPP has been previously established [14], [15].

The CHIRPP database (1990–2009) was searched for individuals 5–19 years old who presented with a brain injury (“minor closed head injury”, “concussion”, “intracranial injury”) while participating in ice hockey (hockey), soccer, American football (football), basketball, baseball, or rugby. Ringette and lacrosse were excluded because of the paucity of players and brain injuries. The extracted variables included age, sex, year, team sport, treatment (admitted/non-admitted), context (informal versus organized), a narrative description of the injury mechanism, and postal code. Sport context was categorized by organized and informal sport. Informal sport was defined as sport that was not regulated by third-party individuals including referees, coaches, or teachers. In contrast, organized sport was defined as sport in a setting in which formal regulation by a referee, coach, or teacher was present. Postal codes were used to determine community type (rural versus urban) [16]. Specific mechanisms of injury codes were developed by two research assistants (e.g.; “checked into boards from behind” or “hit by pitch while batting”). The specific mechanism of injury was then coded for the first 200 cases for each sport independently by the two research assistants using the developed coding list based on the narrative descriptions provided for each case. After establishing inter-coder agreement, the two research assistants applied the resulting codes to the remaining cases. Once coding was complete, proportions of injuries attributable to the specific mechanistic codes were determined. These specific mechanistic codes were also categorized into one of five categories: ‘struck by player’, ‘struck by object’ (in the environment i.e., net, post), ‘struck by sport implement’ (i.e., ball, stick), ‘struck (playing) surface’, and ‘other’.

A descriptive analysis was conducted where normally distributed continuous variables were presented as means and standard deviations and dichotomous and polychotomous variables presented as proportions with associated 95% confidence intervals [17]. A chi-square test was performed to determine if there was an association between community type and sport context. Results were stratified by sport, sex, age group (5–9, 10–14, and 15–19 years), and helmet use. Helmet use data was analyzed for ice hockey, football, and baseball. Inter-coder reliability was evaluated by calculating chance-corrected Cohen’s Kappa values [18].

## Results

**Figure 1:**
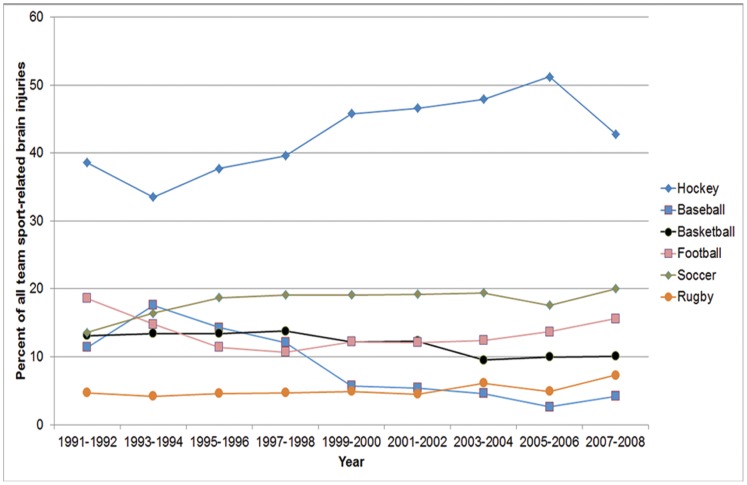
Relative frequencies of brain injuries in each sport by year of incident.

**Figure 2:**
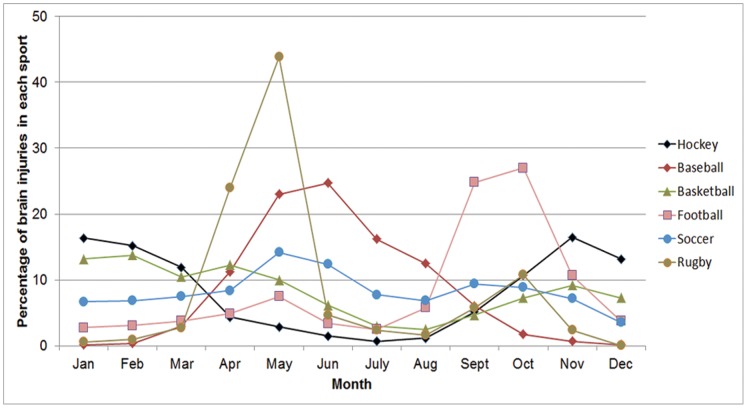
Relative frequencies of brain injuries in each sport by month of incident.

**Figure 3:**
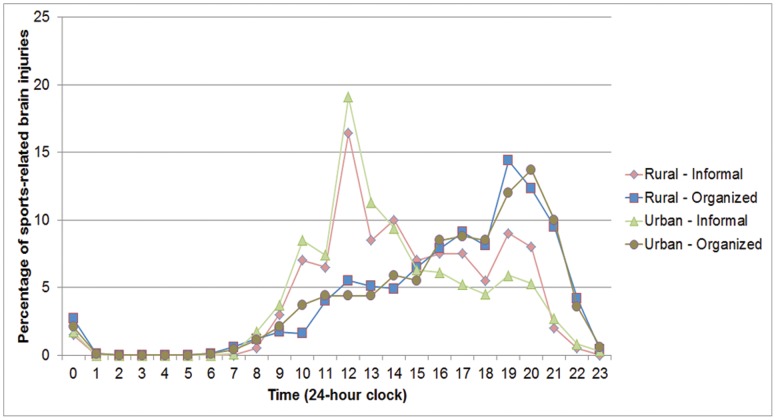
Relative frequencies of brain injuries in each community-context situation by hour of incident.

**Figure 4:**
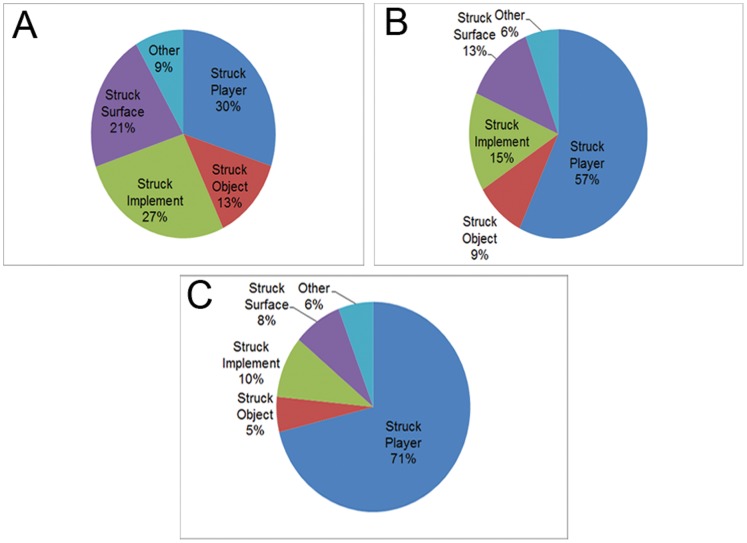
Relative frequencies of brain injury mechanism by age group.

A total of 12,799 team sport brain injuries were identified (16.2% of all CHIRPP reported brain injuries). Hockey accounted for the majority of brain injuries (Figure 1). Overall, 81.4% of the injured players were male and the mean age was 13.2 years (SD 2.8). Each sport had specific months where injuries peaked (Figure 2). Half of all brain injuries occurred during the fall/winter season (October–February: 51.3%), while fewer occurred during the summer (June–August: 13.6%). The highest proportion of brain injuries occurred on weekends (Saturday: 18.2%; Sunday: 17.8%). Approximately half of all brain injuries presented between 16∶00–22∶59 (52.1%). Informal team sports had a peak in the number of brain injuries during the early afternoon and evening; conversely, organized sports had the most number of brain injuries in the early evening (Figure 3). Almost all brain injuries occurred in urban areas (92.2%). Similar proportions of injuries were seen in organized and informal settings within each community type (rural informal: 23.8%; rural organized: 76.2%; urban informal: 25.9%; urban organized: 74.1%; χ^2^ = 2.02, p = 0.16). The proportion of injuries caused by striking another player increased as players aged (Figure 4).

### Ice Hockey

Ice hockey accounted for 5675 (44.3%) of all brain injuries (Table 1) with most occurring among 10–14 year-olds. Being struck by another player was the predominant mechanism (Table 1); specifically, checking into the boards among 10–14 year-olds (males: 36.3%, 95% CI: 34.7–38.0; females: 24.1%, 95% CI: 20.1, 29.6) and 15–19 year-olds (males: 33.2, 95% CI: 30.9, 35.8; females: 26.8%, 95% CI: 21.5, 34.5). Checking from behind resulted in approximately 10% of injuries among 10–14 year-olds (males: 10.6%, 95% CI: 9.7, 11.8; females: 8.3%, 95% CI: 6.2, 12.6) and 15–19 year-olds (males: 9.6, 95% CI: 8.3, 11.4%; females: 9.8%, 95% CI: 7.0, 14.5). The 5–9 year-old group was characterized by falls (males: 34.7%, 95% CI: 31.0, 39.0; females: 48.8%. 95% CI: 37.0, 65.0). Helmet usage was lowest among younger hockey players (70.4%; 95% CI: 66.7, 74.0) and increased to 78.6% (95% CI: 77.2, 79.9) among 10–19 year-olds. Being struck by a player was the most common mechanism of injury in both helmeted and non-helmeted players (Table 2).

**Table 1:** Proportions and 95% CI of brain injury mechanisms among team sports organized by age between 1990–2009 in the Canadian Hospitals Injury Reporting and Prevention Program.

	N	5–9 years oldN (%; 95% CI)	10–14 years oldN (%; 95% CI)	15–19 years oldN (%; 95% CI)
**Ice Hockey**	5 675	591	3543	1541
Struck by player		225 (38.1; 34.5, 42.3)	2293 (64.7; 63.2, 66.3)	1074 (69.7; 67.4, 72.0)
Struck by object		101 (17.1; 14.5, 20.6)	389 (11.0; 10.0, 12.1)	133 (8.6; 7.4, 10.3)
Struck by sport implement		37 (6.3; 4.9, 8.9)	236 (6.7; 5.9, 7.6)	112 (7.3; 6.2, 8.8)
Struck surface		129 (21.8; 18.9, 25.6)	297 (8.4; 7.6, 9.4)	101 (6.6; 5.5, 8.0)
Other		99 (16.8; 14.2, 20.3)	328 (9.3; 8.4, 10.3)	121 (7.9; 6.7, 9.4)
**Soccer**	2 435	543	1274	618
Struck by player		183 (33.7; 30.1, 38.0)	609 (47.8; 45.2, 50.6)	419 (67.8; 64.1, 71.5)
Struck by object		68 (12.5; 10.3, 15.9)	94 (7.4; 6.2, 9.1)	17 (2.8; 2.0, 4.7)
Struck by sport implement		120 (22.1; 19.1, 26.1)	315 (24.7; 22.6, 27.3)	118 (19.1; 16.4, 22.7)
Struck surface		148 (27.3; 23.9, 31.4)	224 (17.6; 15.7, 19.9)	54 (8.7; 7.0, 11.6)
Other		24 (4.4; 3.3, 6.9)	32 (2.5; 1.9, 3.7)	10 (1.6; 1.1, 3.4)
**Football**	1 651	112	965	574
Struck by player		50 (44.6; 36.8, 54.8)	721 (74.7; 71.9, 77.4)	483 (84.2; 81.0, 87.0)
Struck by object		25 (22.3; 16.8, 32.3)	73 (7.6; 6.2, 9.6)	18 (3.1; 2.3, 5.3)
Struck by sport implement		4 (3.6; 2.4, 11.0)	24 (2.5; 1.8, 3.9)	15 (2.6; 1.9, 4.7)
Struck surface		29 (25.9; 19.9, 36.0)	124 (12.9; 11.1, 15.3)	38 (6.6; 5.1, 9.3)
Other		4 (3.6; 2.4, 11.0)	23 (2.4; 1.8, 3.8)	20 (3.5; 2.5, 5.7)
**Basketball**	1 482	151	920	411
Struck by player		39 (25.8; 20.4, 34.3)	378 (41.1; 38.1, 44.4)	245 (59.6; 55.0, 64.4)
Struck by object		19 (12.6; 9.2, 20.7)	90 (9.8; 8.2, 12.1)	28 (6.8; 5.1, 10.2)
Struck by sport implement		34 (22.5; 17.5, 30.9)	171 (18.6; 16.4, 21.4)	34 (8.3; 6.4, 11.8)
Struck surface		51 (33.8; 27.6, 42.9)	260 (28.3; 25.6, 31.4)	98 (23.8; 20.3, 28.6)
Other		8 (5.3; 3.6, 11.6)	21 (2.3; 1.7, 3.7)	6 (1.5; 1.0, 3.8)
**Baseball**	835	280	443	112
Struck by player		13 (4.6; 3.3, 8.6)	46 (10.4; 8.2, 14.0)	20 (17.9; 13.1, 27.5)
Struck by object		3 (1.01; 0.1, 4.9)	7 (1.6; 1.1, 3.8)	4 (3.6; 2.4, 11.0)
Struck by sport implement		256 (91.4; 87.7, 94.2)	366 (86.6; 78.9, 85.9)	77 (68.8; 60.3, 77.0)
Struck surface		1 (0.4; 0.4, 3.1)	7 (1.6; 1.1, 3.8)	2 (1.8; 1.4, 8.7)
Other		7 (2.5; 1.7, 6.0)	17 (3.8; 2.8, 6.6)	9 (8.0; 5.5, 16.5)
**Rugby**	721	5	175	541
Struck by player		2 (40.0; 25.1, 84.2)	147 (84.0; 78.1, 88.8)	464 (85.8; 82.6, 88.5)
Struck by object		0	0	5 (0.9; 0.6, 2.6)
Struck by sport implement		0	2 (1.1; 0.9, 5.7)	3 (0.6; 0.4, 2.1)
Struck surface		2 (40.0; 25.1, 84.2)	12 (6.9; 4.8, 12.8)	14 (2.6; 1.8, 4.7)
Other		1 (20.0; 15.8, 75.0)	14 (8.0; 5.6, 14.2)	55 (10.2; 8.2, 13.4)

**Table 2:** Proportions and 95% CI of brain injury mechanisms among team sports organized by protective equipment use between 1990–2009 in the Canadian Hospitals Injury Reporting and Prevention Program.

	N	HelmetN (%; 95% CI)	No protective equipmentN (%; 95% CI)	Other protective equipmentN (%; 95% CI)	UnspecifiedN (%; 95% CI)
**Ice Hockey**	5675	4412 (77.7)	99 (1.7)	508 (9.0)	656 (11.6)
Struck by player		2965 (67.2; 65.8, 68.6)	18 (18.2; 13.2, 28.6)	276 (54.3; 50.2, 58.8)	333 (50.8; 47.1, 54.7)
Struck by object		463 (10.5; 9.7, 11.5)	10 (10.1; 6.9, 19.6)	87 (17.1; 14.4, 21.0)	63 (9.6; 7.8, 12.4)
Struck by sport implement		255 (5.8; 5.2, 6.6)	27 (27.3; 20.8, 38.2)	44 (8.7; 6.8, 11.8)	59 (9.0; 7.3, 11.7)
Struck surface		349 (7.9; 7.2, 8.8)	24 (24.2; 18.2, 35.1)	56 (11.0; 8.9, 14.4)	98 (14.9; 12.7, 18.1)
Other		380 (8.6; 7.9, 9.5)	20 (20.2; 14.9, 30.8)	45 (8.9; 7.0, 12.0)	103 (15.7; 13.4, 19.0)
**Football**	1651	713 (43.2)	212 (12.8)	67 (4.1)	659 (39.9)
Struck by player		660 (92.6; 90.4, 94.3)	129 (60.8; 54.5, 67.5)	62 (92.5; 84.1, 96.9)	403 (61.2; 57.5, 64.9)
Struck by object		3 (0.4; 0.3, 1.6)	32 (15.1; 11.6, 21.4)	0 (0)	81 (12.3; 10.3, 15.3)
Struck by sport implement		8 (1.1; 0.8, 2.6)	11 (5.2; 3.6, 10.1)	2 (3.0; 2.3, 14.0)	22 (3.3; 2.5, 5.4)
Struck surface		16 (2.2; 1.6, 3.9)	36 (17.0; 13.2, 23.4)	2 (3.0; 2.3, 14.0)	137 (20.8; 18.1, 24.3)
Other		26 (3.6; 2.7, 5.6)	4 (1.9; 1.3, 6.0)	1 (1.5; 1.5, 12.0)	16 (2.4; 1.7, 4.3)
**Baseball**	835	56 (6.7)	106 (12.7)	54 (6.5)	619 (74.1)
Struck by player		11 (19.6; 13.6, 34.7)	8 (7.5; 5.1, 16.2)	12 (22.2; 15.5, 37.7)	48 (7.8; 6.2, 10.5)
Struck by object		1 (1.8; 1.8, 14.1)	1 (0.9; 1.0, 7.9)	1 (1.9; 1.8, 14.6)	11 (1.8; 1.2, 3.6)
Struck by sport implement		41 (73.2; 61.6, 83.7)	90 (84.9; 77.3, 90.7)	39 (72.2; 60.4, 83.0)	529 (85.5; 82.5, 88.1)
Struck surface		1 (1.8; 1.8, 14.1)	2 (1.9; 1.5, 9.1)	1 (1.9; 1.8, 14.6)	6 (1.0; 0.7, 2.5)
Other		2 (3.6; 2.7, 16.4)	5 (4.7; 3.2, 12.8)	1 (1.9; 1.8, 14.6)	25 (4.0; 3.0, 6.3)

### Soccer

Soccer resulted in 2435 (19.0%) brain injuries (Table 1), with most occurring among 10–14 year-olds. Being struck by another player was one of the main mechanisms (Table 1), especially in the form of collisions (including head-to-head collisions) among 10–14 year-olds (males: 21.9%, 95% CI: 19.4, 25.1; females: 23.1%, 95% CI: 19.8, 27.4) and 15–19 year-olds (males: 37.6%, 95% CI: 32.7, 43.6; females: 31.3%, 95% CI: 26.8, 37.0). Injury due to the ball was common among 5–9 year-olds (males: 20.6%, 95% CI: 17.5, 24.9; females: 29.9%, 95% CI: 22.8, 41.7) and 10–14 year-olds (males: 21.3%, 95% CI: 18.8, 24.5; females: 30.5%, 95% CI: 26.8, 35.1). Players 5–9 years old had a greater proportion of injuries due to striking an object in the environment, such as the net or post (Table 1). Females 15–19 years old had a greater number of injuries than males of the same age group. A greater proportion of females were injured by the ball than males (females: 26.6%, 95% CI: 24.0, 29.8; males: 17.2%, 95% CI: 15.5, 19.3). Brain injuries from kicks to the head increased with age (5–9 year-olds: 2.2%, 95% CI: 1.5, 4.3; 15–19 year-olds: 9.7%, 95% CI: 7.9, 12.6).

### Football

Football was the third leading cause of brain injuries (N = 1,651, 12.9%; Table 1) with most occurring among 10–14 year-olds. Striking another player was the main mechanism of injury (Table 1). Specifically, tackling was the predominant mechanism and increased with age. The second most common mechanism was non-tackling collision/contact with other players and also increased with age, particularly head-to-head/body collisions among 15–19 year-olds (males: 25.3%, 95% CI: 22.1, 29.4; females: 12.5%, 95% CI: 8.4, 33.5). Injuries due to striking an object in the environment were most frequent among 5–9 year-olds (Table 1). Helmet use increased from 12.5% (95% CI: 8.8, 21.6) among 5–9 year-olds to 57.5% (95% CI: 53.6, 61.6) among 15–19 year-olds. Regardless of helmet status, being struck by a player was the most common mechanism (Table 2).

### Basketball

Basketball resulted in 1,482 (11.6%) brain injuries (Table 1) with the greatest number occurring among 10–14 year-olds. The proportions of injuries due to collision/contact with other players increased with age, especially elbowing (5–9 year-olds: 0%; 15–19 year-olds: 11.2%, 95% CI: 8.9, 15.1). Striking an object in the environment occurred most often among 5–9 year-olds (Table 1).

### Baseball

Baseball resulted in 835 (6.5%) brain injuries (Table 1) with most occurring among 10–14 year-olds. The most common mechanism in 10–19 year-olds was being hit by the baseball (males: 58.0%, 95% CI: 53.4, 63.0; females: 70.1%, 95% CI: 62.9, 77.0). Being hit by the bat was common among 5–9 year-olds (males: 53.8%, 95% CI: 47.9, 60.5; females: 60.9%, 95% CI: 48.4, 74.8) and often due to being too close to the batter (males: 26.1%, 95% CI: 21.5, 32.7; females: 28.3%, 95% CI: 20.0, 45.3). The proportion of brain injuries caused by player collisions increased with age, but only 0.6% of brain injuries were a result of sliding collisions. Overall, 6.7% of players wore a helmet, and helmet use increased to 15.2% of 15–19 year-olds. Being struck by an implement was the most common mechanism among helmeted and non-helmeted players (Table 2).

### Rugby

There were 721 (5.6%) brain injuries in rugby (Table 1) with most occurring among 15–19 year-olds. Striking another player was the main mechanism of injury (Table 1). More specifically, the main mechanisms in this age group were being tackled or tackling another player (males: 48.5%, 95% CI: 43.7, 53.9; females: 51.7%, 95% CI: 45.0, 59.4), head-to-head collisions (males: 10.2%, 95% CI, 7.9, 14.3; females: 7.3%, 95% CI, 5.1, 13.3 ), and head-to-knee collisions (males: 9.1%, 95% CI, 7.0, 13.0; females: 5.1%, 95% CI, 3.5, 10.6).

### Reliability

Cohen’s Kappa values calculated for each sport were greater than 0.87 (range: 0.87 to 0.95) and interpreted as almost perfect agreement [19].

## Discussion

The majority of team sport brain injuries occurred among males, between October and February, and among hockey players (likely due to the high participation in Canada). A large number of individuals who suffered injuries in informal sports injuries presented to the ED in the early afternoon. This may be due to injuries sustained on the school yard during recess and after school. In hockey, checking into the boards and falls were the most common mechanisms for players older than and younger than 10 years old, respectively. Soccer-related injuries among older players were characterized by player collisions and kicks to the head, while younger children tended to be struck by an object in the environment (e.g., goal post). In football, tackling was the predominant mechanism and similar findings have been observed elsewhere [12]. In basketball, proportions of injuries due to player collisions/contact including elbowing increased with age. In football and basketball, younger players tended to strike fixed structures. In baseball, the main mechanism was being struck by the baseball or bat. Injuries suffered by 5–9 year-olds were characterized mainly by being hit by the bat, often because of standing too close to the batter. Rugby was mainly characterized by player collisions.

Our research provides a baseline level of understanding for mechanisms of injury and highlights important areas of concern for the prevention of team sport-related brain injuries. Body checking has previously been identified as a risk factor for ice hockey injury [4], [20], [21]. We found that it was specifically checking into the boards that caused the greatest proportion of brain injuries, and that 10% of brain injuries were caused by checking into the boards from behind. This is despite rules prohibiting this action for the past twenty years. Our results support increased efforts to completely remove checking from behind. Leagues that disallow body-checking have demonstrated a significant reduction in injuries, and our findings further support the idea that making these manoeuvres illegal has the potential to reduce injuries [20]. In soccer, enforcement of stricter penalties for high kicks may reduce the frequent number of injuries due to kicks to the head. Similar efforts to reduce player-to-player intentional contact in football, rugby, basketball and soccer may also provide benefit.

Education is an important component of a multifaceted approach to injury prevention in sports. Such education should not only manifest itself as general information about brain injury, but also include very sport-specific messaging that addresses the common mechanisms of brain injury in specific sports. This education should be mandatory to participate in the organized sport and be aimed at players, coaches, trainers, officials, and parents at all levels. Furthermore, education can also aim to support rule changes in sports that may help to reduce injuries such as reducing body checking [4]. Skill improvement such as teaching younger hockey players proper skating technique could diminish fall-related brain injuries. In soccer, education about high kicks, scissor kicks, and heading the ball in close proximity to other players should be key targets for education. In baseball, education around creating safe distances from a batter, and mandatory helmets, especially for younger players is important. A systematic review in rugby shows that such educational efforts should be universally mandated and be combined with other efforts such as rule changes to be most effective [22].

Equipment and environmental changes are important avenues for prevention of youth brain injuries. Proper and mandatory universal helmet use and properly supervised no-stand zones for young and recreational baseball players hold significant promise for injury reduction. Making all goal posts and fixed structures in the vicinity of playing surfaces padded and mobile so as to lessen impact forces could reduce brain injury in soccer, basketball, and possibly hockey as well. In rugby, mouth guards and headgear are of limited benefit in reducing neurological injuries [22]; more research is needed to determine whether head protectors would reduce brain injuries in soccer and basketball. We found that helmeted and non-helmeted players were injured by similar mechanisms in ice hockey, football, and baseball, suggesting that other modalities of prevention need to be instituted in addition to personal protective equipment.

### Implications

Our results provide a basis for the modification of existing programs to incorporate multifaceted approaches addressing universal rule changes, safer playing environments, personal protective equipment use, education and economic programs. Some states have introduced laws to mandate such changes [23], but this strategy’s effectiveness has not been evaluated. Economic incentives, such as improved insurance rates for sports with improved safety, should also be explored. As brain injury occurrence comes to greater public awareness, lawsuits that demand accountability from organizations and leagues will create pressure for change. Monitoring systems for the frequency/pattern of injuries should be implemented at a national or provincial level. Independent bodies that can monitor brain injury rates and efforts to reduce them should be established at the national or provincial level of all organized sports. Such bodies should be accountable to a higher level of national audit and have the authority and resources to implement changes to make that particular sport as safe as possible for as many participants as possible. Further research is needed to understand the mechanisms of traumatic brain injury in sports.

### Limitations

This study had several limitations. Firstly, the total number of youth playing each sport was unavailable thus sport, sex, age, or mechanism-specific injury rates were not calculated. As of 2010, Statistics Canada reported that there were 5 616 700 children in Canada aged 14 and under [25]. The Canadian Fitness and Lifestyle Research Institute found that in the year between March 2010 and April 2011, 38% of Canadian children aged 5–17 played soccer within the last year, followed by hockey/ringette (23%), swimming (17%), basketball (15%), and baseball (10%) [24]. This means that roughly 2 000 000 played soccer, 1 300 000 played hockey/ringette, 840 000 played basketball, and 560 000 played baseball in 2010. This indicates that many children are at risk for suffering sport-related traumatic brain injury and that appropriate understanding of how they are getting injured is important to prevent these injuries. In this regard, more consistently available information on sports participation is essential to aid future research. This information can help with calculating injury rates but also aid in injury surveillance and the evaluation of injury prevention strategies.

The CHIRPP database is limited to brain injuries presenting to EDs so any injuries that did not present to the ED would have been missed. Fatal injuries were also not included in CHIRPP, but pediatric sport-related fatalities are rare [26], and a recent paper has further demonstrated the representativeness of CHIRPP for injury profiling [27]. Additionally, the mechanism of injury was provided in a text field and had to be coded by two individuals. Some narratives where unclear; however, the individuals consulted each other on unclear cases. A descriptive analysis was performed without multivariate modelling; however, the results were stratified by age groups and sex. Although there may be residual confounding by age group since the effect of exact age was not examined, previous papers have stratified by age as we did in the current paper [10], [11].

Additionally, there may certainly be differences depending on the position being played; however, positional information was not consistently available in CHIRPP. As a result, we were unable to resolve these differences in this study. Future studies should focus on differences in the frequencies, proportions, and severity of injuries sustained in varying positions within specific sports.

### Conclusion

This study is the first to comprehensively analyze mechanisms of injury of pediatric team sports brain injuries. Intervention should be aimed at educating players, the public, and sport organizations about the most common mechanisms of injuries. Differences in mechanisms across sports and ages should help tailor future prevention efforts. Ultimately, a multifaceted approach in all sports holds promise for prevention of brain injury.

## References

[1] Warburton DE, Nicol CW, Bredin SS (2006) Health benefits of physical activity: the evidence. CMAJ 174: 801–809. 16534088 10.1503/cmaj.051351 PMC1402378

[2] Langlois JA, Rutland-Brown W, Wald MM (2006) The epidemiology and impact of traumatic brain injury: a brief overview. J Head Trauma Rehabil 21: 375–378. 16983222 10.1097/00001199-200609000-00001

[3] Benz B, Ritz A, Kiesow S (1999) Influence of age-related factors on long-term outcome after traumatic brain injury (TBI) in children: a review of recent literature and some preliminary findings. Restor Neurol Neurosci 14: 135–141. 22387509

[4] Macpherson A, Rothman L, Howard A (2006) Body-checking rules and childhood injuries in ice hockey. Pediatrics 117: e143–e147. 16452323 10.1542/peds.2005-1163

[5] Emery CA, Meeuwisse WH (2006) Injury rates, risk factors, and mechanisms of injury in minor hockey. Am J Sports Med 34: 1960–1969. 16861577 10.1177/0363546506290061

[6] Juhn MS, Brolinson PG, Duffey T, Stockard A, Vangelos ZA, et al (2002) Position statement: Violence and injury in ice hockey. Clin J Sport Med 12: 46–51. 11854591 10.1097/00042752-200201000-00014

[7] Goodman D, Gaetz M, Meichenbaum D (2001) Concussions in hockey: there is cause for concern. Med Sci Sports Exerc 33: 2004–2009. 11740291 10.1097/00005768-200112000-00005

[8] Emery CA, Hagel B, Decloe M, Carly M (2009) Risk factors for injury and severe injury in youth ice hockey: as systematic review of the literature. Inj Prev 16: 113–118. 10.1136/ip.2009.022764 20363818

[9] Delaney JS (2004) Head injuries presenting to emergency departments in the United states from 1990 to 1999 for ice hockey, soccer, and football. Clin J Sport Med 14: 80–87. 15014341 10.1097/00042752-200403000-00005

[10] Kelly KD, Lissel HL, Rowe BH, Vincenten JA, Voaklander DC (2001) Sport and recreation-related head injuries treated in the emergency department. Clin J Sport Med 11: 77–81. 11403118 10.1097/00042752-200104000-00003

[11] Giannotti M, Al-Sahab B, McFaull S, Tamim H (2010) Epidemiology of acute head injuries in Canadian children and youth soccer players. Injury 41: 907–912. 19878944 10.1016/j.injury.2009.09.040

[12] Delaney JS, Puni V, Rouah F (2006) Mechanisms of injury for concussions in university football, ice hockey, and soccer: a pilot study. Clin J Sport Med 16: 162–165. 16603887 10.1097/00042752-200603000-00013

[13] Public, Health, Agency, of, Canada Canadian Hospitals Injury Reporting and Prevention Program (CHIRPP).

[14] Pickett W, Brison RJ, Mackenzie SG, Garner M, King MA, et al (2000) Youth injury data in the Canadian Hospitals Injury Reporting and Prevention Program: do they represent the Canadian experience? Inj Prev 6: 9–15. 10728534 10.1136/ip.6.1.9 PMC1730589

[15] Macarthur C, Dougherty G, Pless IB (1997) Reliability and validity of proxy respondent information about childhood injury: an assessment of a Canadian surveillance system. Am J Epidemiol 145: 834–841. 9143214 10.1093/oxfordjournals.aje.a009177

[16] Hassan A, Pearce NJ, Mathers J, Veugelers PJ, Hirsch GM, et al (2009) The effect of place of residence on access to invasive cardiac services following acute myocardial infarction. Can J Cardiol 25: 207–212. 19340343 10.1016/s0828-282x(09)70062-5 PMC2706757

[17] Agresti A, Coull BA (1998) Approximate is better than ‘exact’ for interval estimation of binomial proportions. Am Stat 52: 119–126.

[18] Cohen J (1960) A coefficient of agreement for nominal scales. Educ Psychol Meas 20: 37–46.

[19] Landis JR, Koch GG (1977) The measurement of observer agreement for categorical data. Biometrics 33: 159–174. 843571

[20] Emery CA, Kang J, Shrier I, Goulet C, Hagel BE, et al (2010) Risk of injury associated with body checking among youth ice hockey players. JAMA 303: 2265–2272. 20530780 10.1001/jama.2010.755

[21] Cusimano MD, Taback NA, McFaull SR, Hodgins R, Bekele TM, et al (2011) Effect of bodychecking on rate of injuries among minor hockey players. Open Med 5: e57–65. 22046222 PMC3205817

[22] Cusimano M, Nassiri F, Chang Y (2010) The effectiveness of interventions to reduce neurological injuries in rugby union. Neurosurgery 67: 1404–1418. 20871440 10.1227/NEU.0b013e3181f209f1

[23] Injury TB Zackery Lystedt Law - House Bill 1824.

[24] Canadian Fitness & Lifestyle Research Institute (2011) Getting Kids Active! 2010 Physical Activity Monitor: Facts and Figures.

[25] Statistics Canada, Section 2: Age and sex (2010). Available: http://www.statcan.gc.ca/pub/91-215-x/2010000/part-partie2-eng.htm.Accessed 12 January 2013.

[26] Turk EE, Riedel A, Püeschel K (2008) Natural and traumatic sports-related fatalities: a 10-year retrospective study. Br J Sports Med 42: 604–208. 17761785 10.1136/bjsm.2007.038505

[27] Kang J, Hagel B, Emery CA, Senger T, Meeuwisse W (2012). Assessing the representativeness of Canadian Hospitals Injury Reporting and Prevention Programme (CHIRPP) sport and receational injury data in Calgary, Canada. Int J Inj Contr Saf Promot 1–8. 10.1080/17457300.2012.656315 22364113

